# Chemical Degradation of Methylene Blue Dye Using TiO_2_/Au Nanoparticles

**DOI:** 10.3390/nano11061605

**Published:** 2021-06-18

**Authors:** Luiza Izabela Jinga, Gianina Popescu-Pelin, Gabriel Socol, Sorin Mocanu, Madalina Tudose, Daniela C. Culita, Andrei Kuncser, Petre Ionita

**Affiliations:** 1 Faculty of Chemistry, University of Bucharest, 90 Panduri, 050663 Bucharest, Romania; izabela.jinga@inflpr.ro; 2 National Institute for Lasers, Plasma and Radiation Physics, 077125 Magurele, Romania; gianina.popescu@inflpr.ro (G.P.-P.); gabriel.socol@inflpr.ro (G.S.); 3 Institute of Physical Chemistry, 202 Spl. Independentei, 050663 Bucharest, Romania; sorin.mocanu89@gmail.com (S.M.); danaculita@yahoo.co.uk (D.C.C.); 4 National Institute of Materials Physics, 077125 Magurele, Romania; akuncser@yahoo.com

**Keywords:** environmental remediation, nanoparticle, hydrogen peroxide, gold, titania

## Abstract

Gold nanoparticles (~10 nm) were deposited on titanium dioxide nanoparticles (~21 nm) and the material obtained was characterized using IR, UV-Vis, N_2_ adsorption–desorption isotherm, DLS, EDS (EDX), TEM, XPS, and XRD techniques. It was found that the methylene blue dye is degraded in the presence of this material when using hydrogen peroxide as the oxidant. Tests were performed at 2, 4, 6, and 24 h, with hydrogen peroxide contents varying from 1 to 5 mg/mL. Longer exposure time and a higher content of oxidant led to the degradation of methylene blue dye at up to 90%. The material can be reused several times with no loss of activity.

## 1. Introduction

Organic pollutants are largely encountered in both water sources and wastewaters. Removal of such contaminants is possible through several processes, including physical operations (adsorption), biological degradation (facilitated by microorganisms), and chemical oxidation (using air or other co-oxidant). All of these methods possess certain advantages, as well as some drawbacks. Depending on the nature of organic pollutants, one or more of such processes are used.

Nowadays, there is keen interest in green processes that are less harmful to the environment. This includes the use of catalysts or photo processes that are cleaner in terms of the chemicals used. Heterogenous photocatalysis involving light, titanium dioxide (TiO_2_), and hydrogen peroxide (H_2_O_2_) is currently one of the best options owing to its low cost and high efficiency [1,2,3]. This process involves the hydroxyl free radical (HO^.^) as the active species, an extremely reactive and unselective agent able to attack even the most stable organic compounds. The hydroxyl free radical is easily produced by photolysis of hydrogen peroxide or in chemical Fenton-like processes favored by transition metal ions. Other similar reactive oxygen species (ROS) are well known for their activity towards organic molecules [4].

It is worth noting that TiO_2_ is a semiconductor [5] used to degrade organic pollutants via oxidative processes—its main role is to activate and make the electron transfers possible. In this way, either the organic pollutant (i.e., a dye) or the oxidant can be activated, further generating a cascade of redox processes that can end with complete mineralization of the organic pollutants. Data from the literature showed that combining such semiconductors with noble metal nanoparticles might lead to an increase in the photocatalytic efficiency [6,7].

Although the real photonic mechanism for the hybrid materials formed from semiconductors and noble metal nanoparticles is not completely understood and depends on many factors, it is believed that the surface plasmon resonance is an important step [8,9]. Regardless, well-documented synthetic protocols are available, allowing good control of the size and dispersion of nanomaterials [10].

Gold nanoparticles (Au NPs) are most used due to their advantages—they are easily obtained with various sizes and morphologies, but most importantly they can be used in extremely large types of reactions—even in opposite reactions, such is oxidation or reduction. Additionally, data from the literature showed that these nanoparticles can be used in hybrid materials [11,12,13,14,15,16]. Moreover, deposited Au NPs demonstrate much more activity than simple gold powder (under the same experimental conditions) [17], meaning that the solid support plays an important role regarding the catalytic activity [18]. Regarding organic pollutants, compounds derived from large-scale industries are frequently encountered. For example, dyes, medicines, halogeno- or nitro-compounds, solvents, oils, pesticides, and others are such common pollutants found in water or soil. Methylene blue (MB) is an organic cationic dye that is heavily used in the textile and paper industry, which also has medicinal applications. Although MB is considered a non-toxic compound, under sunlight it can produce hazardous oxygen singlets (^1^O_2_) that can damage living beings and affect aquatic environments, increasing public health concerns. Abundant data are available in the literature for various systems dealing with MB removal from wastewater; thus, many types of (nano)composites have been tested, including component graphene or graphene-oxide, carbon-activated materials, metal oxides, magnetic nanoparticles, cellulose, and others [19,20,21,22].

**Figure 1:**
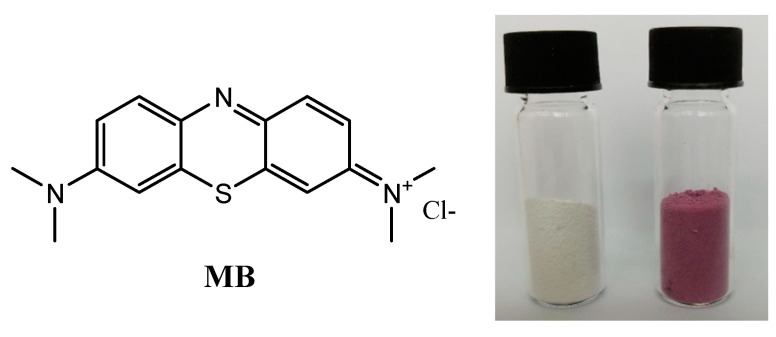
Chemical structure of MB (**left**), titania nanoparticles, and the obtained Au NPs deposited in titania (**right**).

MB is commonly used as a test dye in environmental studies. In this study, we used a hybrid composite of gold nanoparticles deposited on titania (TiO_2_–Au) for degradation of MB (Figure 1).

Many researchers employ mixed nanoparticles, containing noble metal nanoparticles deposited on different kind of supports, due to their facile synthesis method, high activity, ease of recovering and reuse, and numerous applications, ranging from catalytic activity to antibacterial properties [23]. Additionally, the manufacturing of such hybrid materials should not produce environmental pollution and should provide a greener approach.

## 2. Results and Discussion

### 2.1. Material Synthesis and Characterization

Commercially available titania nanoparticles with an average size of 21 nm were used as the support for gold nanoparticles (Au NPs). Au NPs were obtained using the method proposed in [24,25,26]. The deposition was achieved by mixing a solution of Au NPs in dichloromethane (DCM) with titania and evaporating the solvent using a rotaevaporator. In this way, a red-violet solid material (Figure 1) containing about 5% Au was obtained (see experimental section for details). It is well known that the size of the constituent nanoparticles has a tremendous impact on the activity of the material [27]; undoubtedly, the preparation method also plays a crucial role [28].

**Figure 2:**
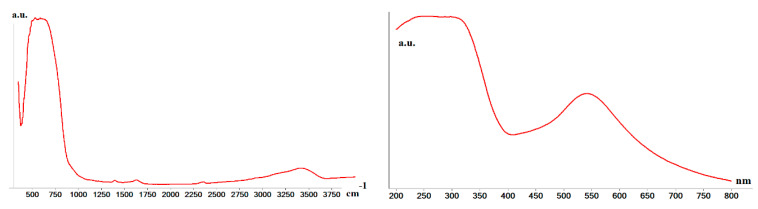
IR (**left**) and UV-Vis (**right**) spectra of the material.

The IR spectrum (Figure 2, left) showed a strong band at 500–750 cm^−1^, commonly known for titania [29]. The UV-Vis spectrum recorded in solid support showed an absorption band at 540 nm (Figure 2, right), which was due to the plasmonic Au NPs [30]. The direct band gap energy *Eg* of 3.45 eV was calculated using the Tauc equation [31], from the intersections of the straight line with the energy axis.

**Figure 3:**
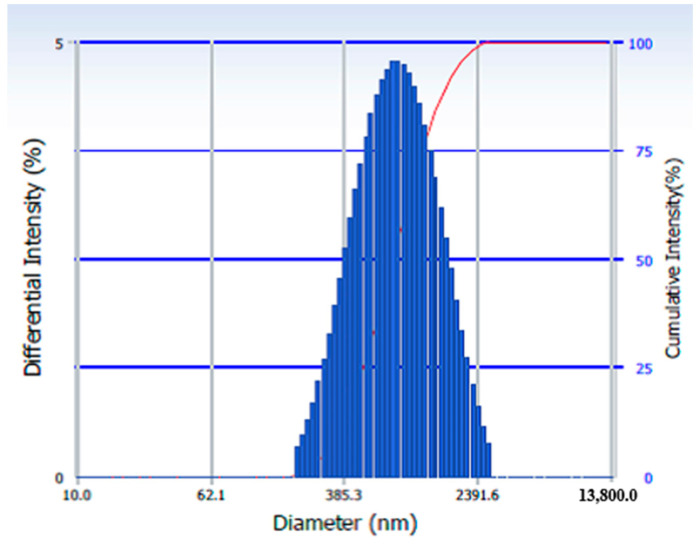
Hydrodynamic diameter distribution determined by DLS analysis.

The size distribution profile of the material nanoparticles suspended in water at 25 °C was determined using dynamic light scattering (DLS) (Figure 3). The hydrodynamic diameter distribution ranged from 200 nm to 2.7 µm, with an average size of 750 nm and a polydispersity index of 0.258. The large difference between the hydrodynamic diameter of the particles and the size determined by TEM (see next) could be attributed to the tendency for agglomeration of suspended particles. Additionally, it should be kept in mind that the hydrodynamic diameter represents the diameter of the particle surrounded by the layers of the solvent that adheres to its surface.

**Figure 4:**
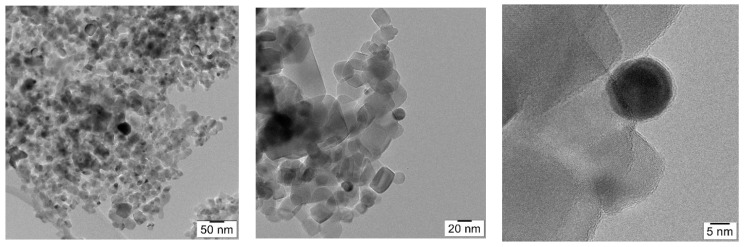
TEM pictures at different magnifications.

Using transmission electron microscopy (TEM), analysis details of the morphology of the material were evidenced. The pictures shown in Figure 4 demonstrate the physical mixture of titania nanoparticles (~21 nm) with gold nanoparticles (~10 nm). The shape and size of both titania and gold nanoparticles can also be noticed. While titania nanoparticles have a square-like shape, gold nanoparticles look more spherical. Additionally, a good dispersion of the gold nanoparticles within the titania was observed.

**Figure 5:**
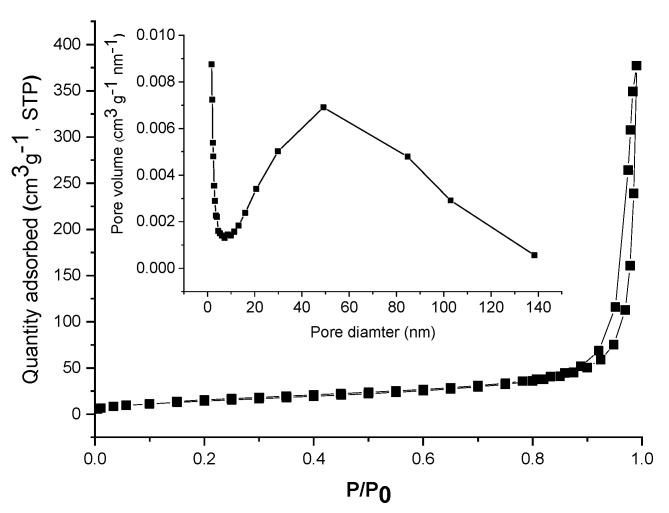
N_2_ adsorption–desorption isotherm of TiO_2_–Au and the corresponding pore size distribution (inset).

Further, the material was subjected to nitrogen adsorption–desorption analysis. The isotherm (Figure 5) was of type IV according to IUPAC classification [32], which is characteristic for mesoporous materials. The specific surface area was determined to be 53.8 m^2^/g, while the total pore volume was 0.583 cm^3^/g. As can be seen from the inset in Figure 5, the pore size distribution is wide, covering the entire mesopore range and even exceeding it. An important part of the porosity is represented by the interparticle voids, as the hysteresis loop appears at high relative pressures.

**Figure 6:**
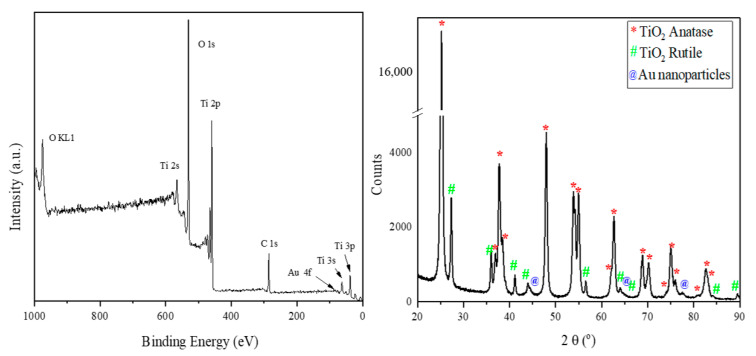
XPS and XRD analyses of the synthesised material.

**Figure 7:**
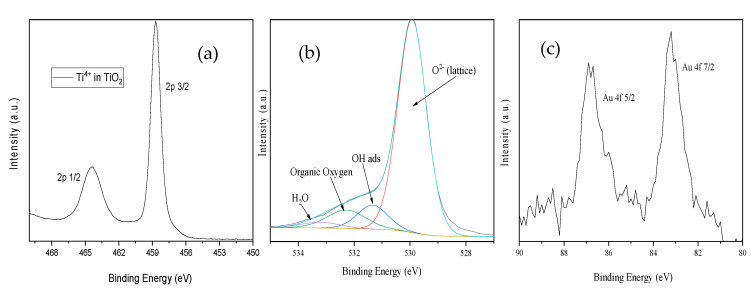
High-resolution XPS spectra: Ti 2p (**a**); the deconvoluted O 1s singlet (**b**); Au 4f (**c**).

For further information about the TiO_2_–Au material, X-ray photoelectron spectroscopy (XPS) and X-ray diffraction (XRD) analyses were performed. The chemical composition of the sample was determined using XPS, showing the presence of Ti, Au, and O, as expected. Figure 6 shows these results, while details of the XPS analysis are shown in Figure 7.

The titanium 2p high-resolution spectrum (Figure 7a) revealed the presence of the Ti^4+^ oxidation state typical for TiO_2_ at binding energies (BEs) of Ti 2p 3/2 = 458.8 eV and 464.3 eV for Ti2p ½ [33]. Additionally, the deconvolution revealed the presence of the Ti^3+^ oxidation state, due to the presence of a reductant medium at Ti 2p 3/2 = 457.1 eV and Ti 2p 1/2 462.9 eV. The deconvoluted high-resolution spectrum of the O1s singlet (Figure 7c) revealed four oxygen chemical species in the BEs region of 535–528 eV; thus, O^2−^ bonded in the TiO_2_ lattice was assigned to O1s at 529.9 eV, while the OH groups and water molecules adsorbed on the surface were detected at 531.5 eV and 533.2 eV, respectively. A small fraction of organic oxygen, originating from the carbon tape, was attributed to O1s located at 532.2 eV. The Au 4f high-resolution spectrum (Figure 7b) with Au 4f7/2 at 83.2 eV and Au 4f 5/2 at 86.78 eV highlighted the presence of the gold metallic nanoparticles (10 nm). It is worth mentioning that the formation of gold metallic NPs is characteristic for binding energies (BEs) shifted towards lower values as compared with Au metallic nanoparticles, which is attributed to the Au 4f7/2 transition around 84 eV [34].

A typical diffractogram pattern for the TiO_2_–Au powder is given in Figure 6 (right). The atanase and rutile phases of TiO_2_ and Au were identified from the experimental XRD pattern using the powder diffraction files 00-064-0863, 00-021-1276, and 03-065-2870, respectively.

The atanase phase was observed by the presence of the main characteristic peaks at 25.2°, 37.7°, and 47.9°, corresponding to planes (101), (004), and (200), respectively; whereas the peaks of the rutile phase were identified at 27.3°, 36.0°, and 54.2°, equivalent to planes (110), (101), and (211), respectively. The main Au peaks were not clearly visible because they were submerged in the TiO_2_ peaks; however, the broadening of peaks at 44.7°, 64.5, and 77.8° could be explained by the presence of gold diffraction planes (200), (220), and (311), respectively.

The phase composition determined after Rietveld refinement was 87.7% anatase, 12.1% rutile, and 0.2% gold. The crystallite sizes for atanase and rutile phases calculated using Scherrer equation were 38.8 and 45.6 nm, respectively.

**Figure 8:**
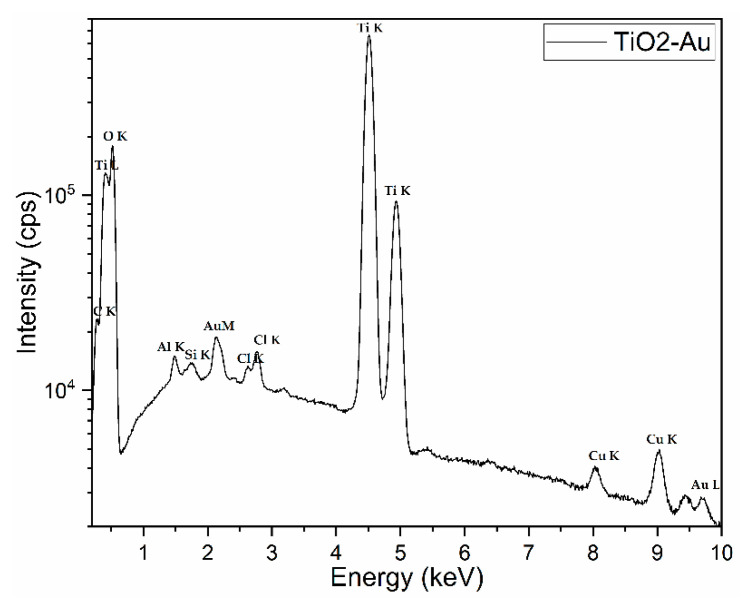
EDX spectrum of the synthesised material.

The chemical composition assessed using EDX spectroscopy is displayed in Figure 8. The EDX spectrum reveals the presence of main elements from the powder, Ti, O, and Au; however, other small peaks for Cl as a residue from the synthetic route and for Al and Cu from the tape and stub used to fix the powder during analysis are shown (peak visible at 2.12 keV) in the synthesized powder. The carbon peak is also visible, which often contaminates the surfaces of samples kept in air.

### 2.2. Application for Removal of MB

The obtained material was tested for the removal of the MB dye, using hydrogen peroxide as the oxidant. Experimental tests demonstrated that hydrogen peroxide alone could not diminish the intensity of the MB color, even at much higher concentrations or over longer times, as compared with those applied in this research. As mentioned before, hydrogen peroxide is one of the most used green oxidants [35,36,37,38].

**Figure 9:**
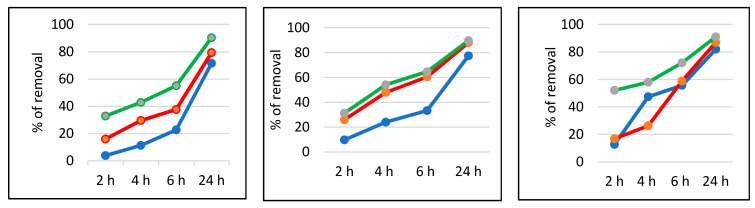
Percentage of removal of MB using 2.5 mg of material (**left**), 5 mg of material (**center**), and 10 mg of material (**right**). Note: hydrogen peroxide contents: 2 mg (blue line), 5 mg (red line), 10 mg (green line).

As a standard work-up, 2.5, 5, or 10 mg of material were suspended in a total of 2 mL MB solution containing either 2, 5, or 10 mg of hydrogen peroxide (see experimental section for details). The mixture was allowed to react for 2, 4, 6, or 24 h and the percentage of removal (%) of the MB dye was calculated using the absorbance of the solution recorded at 663 nm. The results are shown in Figure 9.

As expected, it can be noticed that longer contact times led to higher % of removal, as well as larger quantities of the material or hydrogen peroxide. A recent paper showed that a similar material containing titania and Au NPs or gold nanowires is active in the degradation of several organic dyes, such as MB, rhodamine B, and azophloxine; after five repeated uses, the degradation rate could reach 79–87% [28]. Our experiments regarding the recyclability of the material used in this work showed that under the best conditions (10 mg of material, 10 mg of hydrogen peroxide, and 24 h reaction), there was no loss in the material’s activity after four runs (no further experiments were performed). These results were supported by the high stability of the material, as Au NPs deposited on titania maintain their intrinsic properties.

Regarding the mechanism of action, it is supposed that the TiO_2_–Au nanoparticles act like a Fenton system, yielding the extremely reactive hydroxyl free radical (HO^.^) that is able to degrade the organic molecule of the dye. Therefore, the following reactions are proposed, in accordance with the literature [39]:

H_2_O_2_ + Au^0^ → HO^−^ + Au^+^ + HO (1)

H_2_O_2_ + Au^+^ → H^+^ + Au^0^ + HOO (2)

MB + HO → by-products(3)

To prove the formation of such reactive radicals, the spin-trapping technique was employed, using *t*-butyl-phenylnitrone (PBN) and 5,5-dimethyl-1-pyrroline-*N*-oxide (DMPO) as the spin trap and electron paramagnetic resonance (EPR) as the main technique. While PBN does not affords any EPR signals, in the case of DMPO, very weak signals were recorded (a quartet with an intensity ratio of 1:2:2:1 and with hyperfine coupling constants of *a_N_* = 15 G and *a_H_* = 15 G), which can be attributed without doubt to the HO^.^ free radical [40].

It is necessary to mention that under our working conditions, there were no (or very few) differences between samples run under ambient light or dark conditions. Without Au NPs, no hydroxyl radical is generated from hydrogen peroxide, meaning no fading of the MB is registered. It is well known that the photoelectronic properties of titania are dependent on their surface morphology [41,42]. We also tried to extend the same process to 4-nitrophenolate, an anionic dye (compared with MB, which is a cationic dye), but the results were not reproducible; it is known that some phenol derivatives are types of recalcitrant compounds that can be a serious problem for the environment, as their removal may cause difficulty [43].

## 3. Materials and Methods

Chemicals were purchased from Aldrich, while solvents were purchased from Chimopar and used as received. Titania nanoparticles (average size 21 nm) were an Aldrich product.

Gold nanoparticles were prepared as previously described [25,26] by dissolving 200 mg hydrogen tetrachloroaurate trihydrate in 20 mL double-distilled water; to this solution, 20 mL of toluene containing 320 mg tetraoctylammonium bromide was added. After 5 min of stirring, 460 mg solid triphenylphosphine was added, then stirring continued for 5 min. Then, a solution obtained from 280 mg sodium borohydride and 10 mL water was added and the mixture was stirred for 10 min. The organic phase was separated and the solvent was removed under vacuum. The residue was dissolved in DCM and purified by gel permeation chromatography. The obtained Au NPs, containing about 100 mg gold, were mixed with 2 g of titania and the solvent was slowly evaporated. The solid was heated at 60 °C under vacuum for 2 h, then washed extensively with DCM to remove any trace of organic impurities. UV-Vis spectra were recorded on a JASCO V-670 spectrophotometer (Tokyo, Japan) equipped with solid sample accessories.

IR spectra were recorded on a Jasco FT/IR 4700 using the KBr disk technique (Tokyo, Japan).

Dynamic light scattering (DLS) measurements were performed on aqueous suspensions using a Delsa Nano C particle analyzer (Backman Coulter Brea, Brea, CA, USA).

The nitrogen sorption isotherm was recorded at −196 °C on a Micromeritics ASAP 2020 analyzer (Norcross, GA, USA). The sample was degassed at 200 °C for 4 h under vacuum before analysis. The specific surface area (S_BET_) was calculated according to the Brunauer–Emmett–Teller equation, using adsorption data in the relative pressure range of 0.05–0.30. The total pore volume was calculated from the amount adsorbed at the relative pressure of 0.99. The average pore diameter and pore size distribution curve were obtained using the Barrett–Joyner–Halenda (BJH) method using the desorption branch.

XPS measurements were performed with ESCALAB Xi+ (Thermo SCIENTIFIC Surface Analysis, Waltham MA, USA) equipped with a multichannel hemispherical electron analyzer (dual X-ray source) working with Al Kα radiation (hν = 1486.2 eV). C 1s was used as the energy reference, which has a value of 284.8 eV. The data were recorded on slightly pressed power materials that had been outgassed in the pre-chamber of the setup at room temperature at a pressure of <2 × 10^−8^ Torr to remove the chemisorbed water from their surfaces. The surface chemical compositions and oxidation states were estimated from the XPS spectra by calculating the integral of each peak after subtraction of the “S-shaped” Shirley-type background using the appropriate experimental sensitivity factors using Avantage software (version 5.978). Spectra were analyzed using the NIST X-ray Photoelectron Spectroscopy Database [44,45]. Crystalline phases identification was achieved by X-Ray diffraction (XRD) with an X’Pert Pro MPD diffractometer from Panalytical using Cu Kα radiation (λ = 1.5406 Å) set to work in Bragg–Brentano geometry with 2θ = (20–90)°, a speed of 15 s/step, and 0.02° step.

The elemental composition (in atom %) of TiO_2_–Au powder was assessed by energy dispersive X-ray (EDX) spectroscopy using an EDAX Inc. (Mahwah, NJ, USA) instrument operated at 20 kV. The data were collected from at least four randomly chosen SEM (~150 × 150 µm^2^) areas of the tested powder.

For MB dye degradation, a stock aqueous solution of MB was obtained at a concentration of 0.1 mg/mL. The general settings for the measurements were the following: to 2.5–10 mg of titania-gold material was added a total solution of 2 mL of MB dye, containing 0.02–0.1 mL hydrogen peroxide (10%). The concentration of the remaining MB dye was measured spectrophotometrically at 663 nm. The percentage of removal (%) was calculated using the equation % = (Abs_i_ − Abs_f_)Abs_i_ × 100, where Abs_i_ and Abs_f_ are the initial and final absorbance recorded by the UV-Vis apparatus at 663 nm.

For the reusability test for the material, the measurements were performed using the same procedure, as described; thus, 10 mg of material was added to 2 mL of water containing 0.2 mg MB and 20 mg hydrogen peroxide and the suspension was left at room temperature for 24 h. The next day, the mixture was centrifugated and the solid was reused by again adding 2 mL of water containing 0.2 mg MB and 20 mg hydrogen peroxide and letting the mixture react for another 24 h. The procedure was repeated until the material was reused four times. The concentration of the MB was evaluated spectrophotometrically compared with the solution recovered after centrifugation.

## 4. Conclusions

The material obtained based on titania and gold nanoparticles was characterized by a large number of techniques. It was found that in the presence of hydrogen peroxide, this material could degrade up to 90% of the methylene blue dye. Due to the speed of synthesis, the possibility of recycling, and its low cost, it can be recommended as a promising material for future degradation processes for organic pollutants in the environment.
